# Exacerbating factors in elderly patients with *Mycobacterium avium* complex pulmonary disease

**DOI:** 10.1017/S0950268821000960

**Published:** 2021-04-27

**Authors:** Norio Kodaka, Chihiro Nakano, Takeshi Oshio, Kayo Watanabe, Kumiko Niitsuma, Chisato Imaizumi, Nagashige Shimada, Hirotsugu Morita, Hiroto Matsuse

**Affiliations:** Division of Respiratory Medicine, Department of Internal Medicine, Toho University Ohashi Medical Center, Tokyo, Japan

**Keywords:** Elderly, exacerbating factors, *Mycobacterium avium* complex

## Abstract

No previous studies have examined *Mycobacterium avium* complex pulmonary disease (MAC-PD) in only elderly patients ⩾75 years old. Here, we investigated the exacerbating factors of MAC-PD in elderly patients and clarified cases that can be followed up without MAC medication. From April 2011 to March 2019, 126 advanced aged patients at our institute were newly diagnosed with MAC-PD, and could be observed based on radiological findings for over a year. Their medical records were retrospectively examined for clinical and radiological findings at the time of diagnosis and 1 year later. To identify the predictors of exacerbation, clinical characteristics of 109 treatment-naïve patients were compared between exacerbated and unchanged groups. Additionally, the unchanged group was followed for one more year. In the current study, positive acid-fast bacilli smears from the sputum test, the presence of cavitary lesions and extensive radiological findings, particularly abnormal shadows in ⩾3 lobes, were predictive of exacerbation among treatment-naïve elderly MAC-PD patients. In the unchanged group, <10% showed exacerbation of radiological findings within the subsequent year. In conclusion, if the sputum smear is negative, no cavitary lesions are present, and abnormal shadows are restricted to ⩽2 lobes, elderly patients with MAC-PD may remain untreated for a few years.

## Introduction

The number of patients with *Mycobacterium avium* complex pulmonary disease (MAC-PD) is increasing worldwide [1–4]. With the ageing of populations, an increase in the number of elderly MAC-PD patients is also expected. However, no previous studies have examined MAC-PD in elderly individuals ⩾75 years old. The current study is relevant in Japan because of the high life expectancy. MAC-PD is indolent in nature, and clinical observation without medication is sometimes considered. However, some reports have noted that MAC-PD affects health-related quality of life [5] and that the mortality rate due to MAC-PD is increasing [6–8].

Daley and colleagues assessed the risks and benefits of therapy for MAC-PD [4]. The patient's wishes, the ability to receive treatment and the goals of therapy should be discussed with patients prior to initiating treatment. In some instances, ‘watchful waiting’ may be the preferred course of action [4, 9, 10]. In particular, elderly MAC-PD patients should be carefully evaluated for treatment after considering whether treatment will improve life expectancy and whether side effects including drug interactions are likely to be tolerable [11, 12].

One of the therapeutic agents used to treat MAC-PD, clarithromycin, is poorly tolerated in the elderly [13]. Moreover, when ethambutol (EB) and rifampicin (RFP) are administered to elderly patients, judgement of optic neuritis caused by EB is sometimes difficult due to underlying ocular symptoms such as glaucoma and cataracts. Occasionally, treatment must be discontinued due to nausea and liver disorders attributable to RFP. Follow-up without treatment not only avoids the risk of side effects, but also reduces medical costs. Thus, the aim of this study was to investigate factors that exacerbate MAC-PD in elderly individuals, and to clarify characteristics of cases that can be followed up without MAC treatment.

## Methods

### Study population and design

**Fig. 1.**
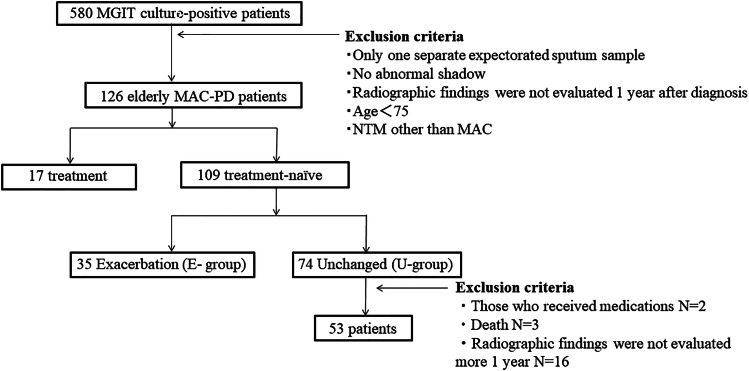
Flow chart of patients diagnosed with *M. avium* complex pulmonary disease between April 2011 and March 2019. MGIT, mycobacterial growth indicator tube; MAC-PD, *M. avium* complex pulmonary disease.

From April 2011 to March 2019 at our institute, of the patients with suspected non-tuberculous mycobacteria (NTM), 580 patients were newly identified by cultures, 251 of whom were newly diagnosed with definitive MAC-PD, according to the 2007 American Thoracic Society/Infectious Disease Society guideline criteria [14]. Those patients <75 years old, those who had no radiographic follow-up after the initial year, and those with NTM other than MAC were excluded. Finally, 126 MAC-PD patients were enrolled in the current study (Fig. 1). The clinical findings of the subjects, including age, sex, laboratory data, past history of tuberculosis, comorbidities and radiological findings, were obtained from their medical records and retrospectively evaluated. To identify the significant predictors of exacerbation and to exclude treatment bias, during the study period, 109 treatment-naïve patients ⩾75 years old who met the inclusion criteria were divided into an exacerbation group (E-group) and an unchanged group (U-group) based on the results of chest computed tomography (CT) 1 year after diagnosis and compared. Additionally, the U-group was followed for one more year.

### Ethics approval and consent to participate

This research was conducted using information previously collected in the course of normal care (without the intention to use it for research at the time of collection). The need for written, informed patient consent was waived in view of the retrospective and observational nature of the study. The study protocol was approved by the Ethics Committee of Toho University Ohashi Medical Center, an ethics committee that reviews research on human subjects (approval no. H20004).

### Microbiological examination

Acid-fast bacilli (AFB) were cultured in a Mycobacteria Growth Indicator Tube from extracted sputum or bronchial washings obtained by bronchoscopy. The sputum samples were obtained on two or more occasions after the initial presentation. The diagnosis of MAC-PD was established when MAC was identified in sputum at least twice or in bronchial washings [4, 14]. MAC was confirmed when cultures were positive for AFB, and the cultured AFB were subsequently confirmed as MAC with polymerase chain reaction.

### Radiological examination and measurements

Chest radiological findings were classified as having a cavity or not on high-resolution CT. Chest radiological findings at the time of initial diagnosis were scored as follows. The lung fields were divided into six lobes based on anatomical structures: right upper; right middle; right lower; left upper (S1 + 2 and S3); left lingular (S4 and S5) and left lower. We assessed whether, at the time of diagnosis, each lung lobe had a shadow such as cavities, bronchiectasis, small nodules, consolidations or atelectasis (maximum six lobes) [15].

CT findings at 1 year after diagnosis were classified into three categories: exacerbation, unchanged or improvement. Each category was defined as follows: exacerbation, abnormal shadows increased; unchanged, abnormal shadows remained stable and improvement, abnormal shadows decreased. The three categories were classified by five board certified members of the Japanese Respiratory Society in a blinded fashion.

### Patient management

When patients did not receive medications for MAC during the observation period, radiographic findings were evaluated at the time of diagnosis and 1 year later. When patients received medications for MAC during the observation period, radiographic findings were evaluated at the start of medications and 1 year later. Patients who received medications for MAC during the observation period (those who began guideline-based therapy and those who discontinued medications) were excluded from the analysis of treatment-naïve subjects.

### Statistical analysis

To identify exacerbating factors related to MAC-PD in treatment-naïve patients, we compared variables in the E-group and U-group using *χ*^2^ tests or Mann–Whitney tests. The performance of the model for predicting exacerbating factors was evaluated using the receiver operating characteristic (ROC) curve by calculating the area under the ROC curve [16–18]. All analyses were performed using SPSS Statistical software (version 22.0; IBM Japan, Tokyo, Japan). *P* values <0.05 were considered significant.

## Results

Table 1 shows the clinical characteristics of the patients and the microbiological and radiological findings. The causative organisms included *M. avium* (97/126, 77.0%), *M. intracellulare* (20/126, 15.9%) and mixed infections (9/126, 7.1%). Their mean age was 82.0 ± 5.59 years, and all patients were over 75 years of age. Females (*n* = 77, 61.1%), never-smokers (*n* = 82, 65.1%), presence of cavitary lesions (*n* = 24, 19.0%) and positive AFB smears (20.6%) were predominant in MAC patients. The mean body mass index (BMI) was low (19.0 ± 3.23 kg/m^2^). The mean number of abnormal lung lobes was 3.22 ± 1.57. Most patients had some kind of comorbidity (115/126, 91.3%).

**Table 1.** Basic characteristics of elderly patients with *M. avium* complex pulmonary disease (*n* = 126)

Age (years)	82.0 ± 5.59
Sex (male/female)	49/77
Species (*M. avium*) (%)	97 (75.8%)
BMI (kg/m^2^)	19.0 ± 3.23
TP (g/dl)	7.30 ± 0.79
ALB (g/dl)	3.47 ± 0.61
CRP (mg/dl)	1.53 ± 3.13
Neutrophil-to-lymphocyte ratio	5.69 ± 6.81
Previous tuberculosis, *n* (%)	14 (11.1%)
Comorbidity, *n* (%)	115 (91.3%)
Positive MAC smear, *n* (%)	26 (20.6%)
Cavitary lesion (%)	24 (19.1%)
Lobes with radiological findings, *n* (%)	3.22 ± 1.57
Smoking history (%)	44 (34.9%)
IgA antibody specific for GPL core antigen^a^	1.9 (1.0–4.3)
Lobes involved ⩾3 (%)	78 (61.9%)
Lobes involved ⩾2 (%)	105 (83.3%)

Data are expressed as mean ± s.d. or numbers (%).

ALB, serum albumin; BMI, body mass index; CRP, serum C-reactive protein; GPL, glycopeptidolipid; MAC, *Mycobacterium avium* complex; TP, serum total protein.

a Reference value only in some cases, IgA antibody specific for GPL core antigen was detected in patients’ serum using a commercially available serodiagnostic kit (Capilia MAC Ab ELISA; Tauns Co.; Ltd., Shizuoka, Japan); lobes involved, number of lobes that had abnormal shadows at the time of diagnosis.

Among the elderly MAC-PD patients, 17 (13.5%) started MAC treatment. Of these 17 patients, during the 1 year after diagnosis, 12 patients were able to continue MAC treatment for more than 6 months (reasons for discontinuation: two cases with suspected EB optic neuritis, one case with nausea, one case with liver damage). Among the treated patients, six showed improvement after treatment, five showed no change after treatment and one showed exacerbation.

**Fig. 2.**
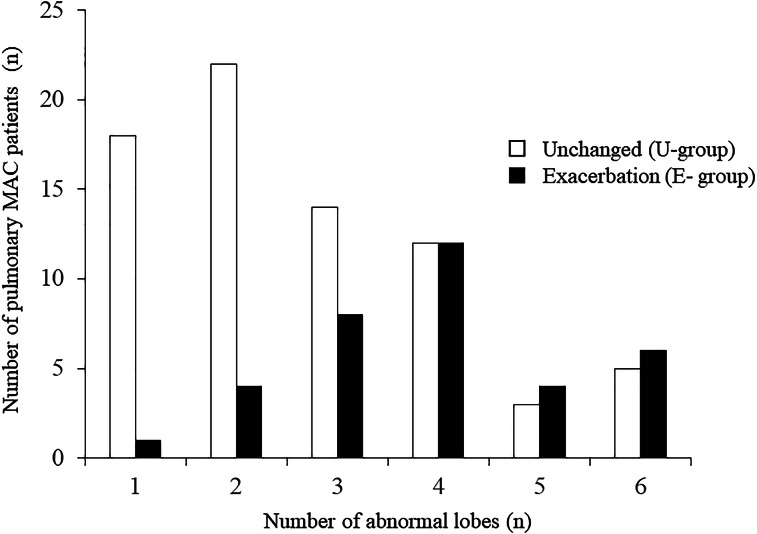
Exacerbation by number of lung lobes with abnormalities. *X*-axis: number of abnormal lobes; *Y*-axis: number of patients with *M. avium* complex pulmonary disease.

To identify significant predictors of exacerbation and to exclude treatment bias, during the study period, 109 treatment-naïve subjects ⩾75 years old met the inclusion criteria and were divided into an E-group (35 patients) and a U-group (74 patients) and compared (Table 2). Positive AFB smears from the sputum test, the presence of cavitary lesions and extensive radiological findings, particularly abnormal shadows in ⩾3 lobes, were predictive of exacerbation among treatment-naïve elderly MAC-PD patients. In patients with lobes showing abnormal findings at the time of diagnosis, 40/45 (88.9%) of those with ⩽2 lobes involved did not show a radiological aggravation in 1 year (Fig. 2).

**Table 2.** Comparison of the characteristics of elderly patients with *M. avium* complex pulmonary disease between the exacerbation group (E-group) and unchanged group (U-group) (*n* = 109)

	E-group (*n* = 35)	U-group (*n* = 74)	*P*-value
Age (years)	81.4 ± 5.56	82.8 ± 5.66	0.17
Sex (female, %)	23 (65.7%)	41 (55.4%)	0.21
Species (*M. avium*) (%)	25 (71.4%)	61 (82.4%)	0.21
BMI (kg/m^2^)	18.7 ± 2.28	19.3 ± 3.61	0.94
TP (g/dl)	7.43 ± 0.66	7.15 ± 0.82	0.05
ALB (g/dl)	3.53 ± 0.69	3.39 ± 0.62	0.49
CRP (mg/dl)	1.17 ± 3.17	1.82 ± 3.31	0.29
Neutrophil-to-lymphocyte ratio	5.24 ± 6.53	6.31 ± 7.60	0.31
Previous tuberculosis, *n* (%)	7 (20%)	6 (8.1%)	0.07
Comorbidity, *n* (%)	31(88.6%)	69 (93.2%)	0.46
Positive MAC smear, *n* (%)	13 (37.1%)	7 (9.5%)	<0.001
Cavitary lesion (%)	11 (31.4%)	8 (10.8%)	0.01
Lobes with radiological findings, *n* (%)	3.91 ± 1.34	2.66 ± 1.46	<0.001
Smoking history	10 (28.6%)	31 (41.9%)	0.21
IgA antibody specific for GPL core antigen^a^	3.8 (0.8–6.2)	1.5 (1.05–3.35)	0.07
Lobes involved ⩾3 (%)	30 (85.7%)	34 (45.9%)	<0.001
Lobes involved ⩾2 (%)	32 (91.4%)	56 (75.7%)	0.07

Data are expressed as mean ± s.d. or numbers (%).

ALB, serum albumin; BMI, body mass index; CRP, serum C-reactive protein; GPL, glycopeptidolipid; MAC, *Mycobacterium avium* complex; TP, serum total protein.

a Reference value only in some cases, IgA antibody specific for GPL core antigen was detected in patients’ serum using a commercially available serodiagnostic kit (Capilia MAC Ab ELISA, Tauns Co., Ltd., Shizuoka, Japan); lobes involved = number of lobes that had abnormal shadows at the time of diagnosis.

**Fig. 3.**
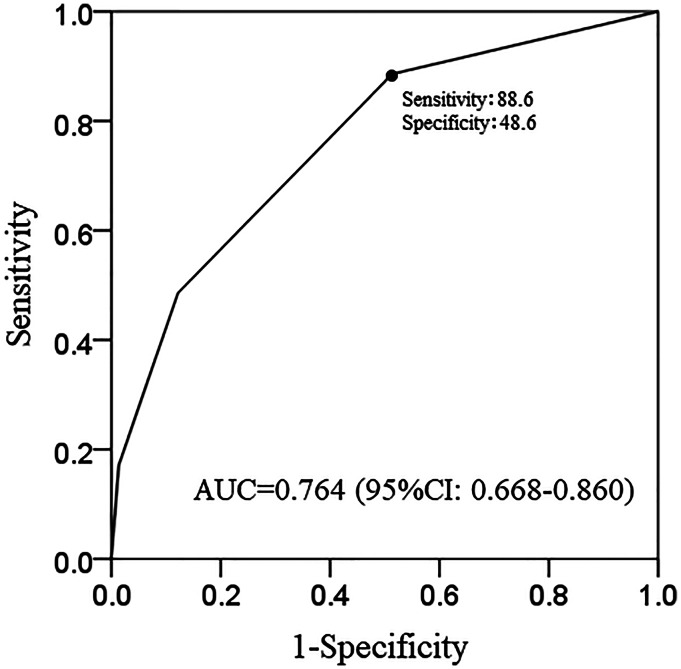
Exacerbation prediction model (simple classification): ROC curve. AUC, area under the curve.

Additionally, the ROC curve analysis of the three factors that were significant predictors of exacerbation (positive AFB smears from the sputum test, presence of cavitary lesions and abnormal shadows in ⩾3 lobes) was performed to determine the threshold value of how many factors conferred a risk of exacerbation. To establish a simple classification of exacerbating factors, in the additional analysis with the ROC curve for the three significant factors, the best cut-off value was 1, which showed a sensitivity of 88.6% and specificity of 48.6%. The ROC curve had an area under the curve of 0.764 for the exacerbation prediction model (Fig. 3).

We further analysed whether the group that did not show exacerbation in 1 year remained unchanged during the subsequent year. In 53 cases in the U-group, the radiological examination for the following year could be confirmed. Forty-nine of 53 cases (92.5%) remained unchanged during the subsequent year.

## Discussion

No studies have examined MAC-PD in elderly individuals ⩾75 years old. Thus, we investigated exacerbating factors in MAC-PD in elderly people, and clarified patients that can be followed up without MAC treatment.

In the current study, of 126 patients, 17 patients started treatment. Although only 12 of 17 patients were able to undergo treatment for more than 6 months, the improvement in treatment was 50% or more. Importantly, treatment may be selected not only for those whose condition is deemed by attending physicians to likely worsen, but also for elderly patients who are resistant or refractory to treatment. For this reason, we decided to study 109 cases excluding the treatment group. In the examination of only elderly MAC-PD patients in our hospital, exacerbation occurred in 35 of 109 (32.1%) patients, which tended to be higher than that of other studies. However, if the start of treatment was defined as exacerbation, 17 of 126 (13.4%) patients experienced exacerbation, and this tended to be low [19].

Disease progression of MAC-PD was defined as either requiring the start of treatment [20, 21] or the presence of aggravation on radiological imaging [22, 23]. In clinical practice, many elderly MAC-PD patients cannot begin MAC treatment due to the presence of other diseases and medications being taken. Thus, treatment may have been selected for patients under good general conditions. In addition, some patients do not wish to receive long-term MAC treatment. Consequently, disease progression in this study was defined as exacerbation seen on radiological imaging. Sometimes, deciding whether or not to start treatment for MAC-PD in the elderly is still very difficult. In fact, only 13.5% of MAC-PD patients in the current study began treatment. This study showed that positive AFB smears from the sputum test, the presence of cavitary lesions and abnormal shadows in ⩾3 lobes were associated with exacerbations based on the probabilities of reactivation or dissemination of the infection. These three factors were in accordance with the findings of a previous study [20]. In other words, if the sputum smear was negative, no cavitary lesions were present, and abnormal shadows were restricted to ⩽2 lobes, elderly MAC-PD patients might not need treatment. At the time of diagnosis, although half of the patients in this study with abnormal findings in ⩾4 lobes did show a radiological exacerbation in 1 year, most of those with ⩽2 lobes involved did not show a radiological exacerbation in 1 year (Fig. 2). To establish a simple classification of exacerbating factors, in the additional analysis with the ROC curve [24, 25] for the three significant factors (positive AFB smears from the sputum test, the presence of cavitary lesions, abnormal shadows in ⩾3 lobes), the best difference was for one or more items, which showed a sensitivity of 88.6% and specificity of 48.6%. In short, in elderly MAC-PD patients 75 years of age and over, the three significant factors increase the risk, and if none of these three factors are found, follow-up can be performed without treatment.

In regular medical treatment, we have encountered many patients with MAC-PD in which the radiological images have not changed over a long time. We further analysed whether the group that did not experience an exacerbation in 1 year remained unchanged during the next year. In 53 cases in the U-group, the radiological examination for the following year could be confirmed. Forty-nine of these 53 cases (92.5%) remained unchanged. All four cases with exacerbation 2 years after diagnosis were classified as having one or more of the factors that were identified in our simple classification using the ROC curve. Thus, our simple classification of the exacerbating factors may be useful for making treatment decisions in elderly patients with MAC-PD.

## Limitations

The current study has several limitations that must be acknowledged. First, this study is limited to elderly patients, and the sample size was relatively small. Second, data collection was retrospective, and thus, sputum cultures, laboratory data and CT scans were obtained according to clinical practice rather than strict schedules. Third, because this investigation targeted elderly MAC-PD patients with many underlying diseases, the results may have been affected by these comorbidities and the therapeutic agents taken to treat them. Finally, 2 years after diagnosis, some patients had died of other diseases and some patients were not followed up. In these cases, we could not determine whether or not MAC-PD had worsened.

Most studies of exacerbating factors of MAC-PD are retrospective, and prospective studies remain limited to small numbers of patients. Because the number of patients with MAC-PD is gradually increasing, we eagerly await large-scale, long-term prospective studies undertaken cooperatively between multiple countries.

## Conclusion

Positive AFB smears from the sputum test, the presence of cavitary lesions and abnormal shadows in ⩾3 lobes were associated with exacerbations in elderly MAC-PD patients. Elderly MAC-PD patients without these factors may not need treatment for a few years.

## Data Availability

The data that support the findings of this study are available on request from the corresponding author. The data are not publicly available due to ethical restrictions.

## References

[1] Prevots DR and Marras TK (2015) Epidemiology of human pulmonary infection with nontuberculous mycobacteria: a review. Clinics in Chest Medicine 36, 13–34. 25676516 10.1016/j.ccm.2014.10.002 PMC4332564

[2] Tan Y (2018) Epidemiology of pulmonary disease due to nontuberculous mycobacteria in Southern China, 2013–2016. BMC Pulmonary Medicine 18, 168. 30413193 10.1186/s12890-018-0728-z PMC6230232

[3] Kendall BA and Winthrop KL (2013) Update on the epidemiology of pulmonary nontuberculous mycobacterial infections. Seminars in Respiratory and Critical Care Medicine 34, 87–94. 23460008 10.1055/s-0033-1333567

[4] Daley CL (2020) Treatment of nontuberculous mycobacterial pulmonary disease: an official ATS/ERS/ESCMID/IDSA clinical practice guideline. Clinical Infectious Diseases 71, 905–913. 32797222 10.1093/cid/ciaa1125 PMC7768745

[5] Asakura T (2018) Health-related QOL of elderly patients with pulmonary *M. avium* complex disease in a university hospital. The International Journal of Tuberculosis and Lung Disease 22, 695–703. 29862956 10.5588/ijtld.17.0433

[6] Morimoto K (2014) A steady increase in nontuberculous mycobacteriosis mortality and estimated prevalence in Japan. Annals of the American Thoracic Society 11, 1–8. 24102151 10.1513/AnnalsATS.201303-067OC

[7] Shu CC (2008) Nontuberculous mycobacteria pulmonary infection in medical intensive care unit: the incidence, patient characteristics, and clinical significance. Intensive Care Medicine 34, 2194–2201. 18648768 10.1007/s00134-008-1221-6

[8] Ito Y (2015) Increasing patients with pulmonary *Mycobacterium avium* complex disease and associated underlying diseases in Japan. Journal of Infection and Chemotherapy 21, 352–356. 25640532 10.1016/j.jiac.2015.01.004

[9] Koh WJ (2017) Outcomes of *Mycobacterium avium* complex lung disease based on clinical phenotype. The European Respiratory Journal 50, 1602503. 28954780 10.1183/13993003.02503-2016

[10] Koh WJ (2017) Mycobacterial characteristics and treatment outcomes in *Mycobacterium abscessus* lung disease. Clinical Infectious Diseases 64, 309–16. 28011608 10.1093/cid/ciw724

[11] Griffith DE (1995) Adverse events associated with high-dose rifabutin in macrolide-containing regimens for the treatment of *Mycobacterium avium* complex lung disease. Clinical Infectious Diseases 21, 594–598. 8527549 10.1093/clinids/21.3.594

[12] Parekh M, Kamelhar D and Schluger N (2012) Nontuberculous mycobacterial infections in older patients. Aging and lung disease: a clinical guide. New York: Springer, pp. 189–199.

[13] Wallace RJ Jr., Brown BA and Griffith DE (1993) Drug intolerance to high-dose clarithromycin among elderly patients. Diagnostic Microbiology and Infectious Disease 16, 215–221. 8477575 10.1016/0732-8893(93)90112-k

[14] Griffith DE (2007) An official ATS/IDSA statement: diagnosis, treatment, and prevention of nontuberculous mycobacterial diseases. American Journal of Respiratory and Critical Care Medicine 175, 367–416. 17277290 10.1164/rccm.200604-571ST

[15] Kodaka N (2020) Predictors of radiological aggravations of pulmonary MAC disease. PLoS One 15, e0237071. 32760104 10.1371/journal.pone.0237071 PMC7410298

[16] Rozenshtein A (2015) Radiographic appearance of pulmonary tuberculosis: dogma disproved. AJR. American Journal of Roentgenology 204, 974–978. 25905930 10.2214/AJR.14.13483

[17] Centor RM and Schwartz JS (1985) An evaluation of methods for estimating the area under the receiver operating characteristic (ROC) curve. Medical Decision Making 5, 149–156. 3841685 10.1177/0272989X8500500204

[18] Park H, Goo JM and Jo C-H (2004) Receiver operating characteristic (ROC) curve: practical review for radiologists. Korean Journal of Radiology 5, 11–18. 15064554 10.3348/kjr.2004.5.1.11 PMC2698108

[19] Kitada S (2012) Long-term radiographic outcome of nodular bronchiectatic *Mycobacterium avium* complex pulmonary disease. The International Journal of Tuberculosis and Lung Disease 16, 660–664. 22410245 10.5588/ijtld.11.0534

[20] Hwang JA (2017) Natural history of *Mycobacterium avium* complex lung disease in untreated patients with stable course. The European Respiratory Journal 49, 1600537. 28275170 10.1183/13993003.00537-2016

[21] Koh WJ (2012) Clinical significance of the differentiation between *Mycobacterium avium* and *Mycobacterium* intracellulare in *M. avium* complex lung disease. Chest 142, 1482–1488. 22628488 10.1378/chest.12-0494

[22] Gochi M (2015) Retrospective study of the predictors of mortality and radiographic deterioration in 782 patients with nodular/bronchiectatic *Mycobacterium avium* complex lung disease. BMJ Open 5, e008058. 10.1136/bmjopen-2015-008058 PMC4538251 26246077

[23] Pan S-W (2017) Microbiological persistence in patients with *Mycobacterium avium* complex lung disease: the predictors and the impact on radiographic progression. Clinical Infectious Diseases 65, 927–934. 28541556 10.1093/cid/cix479

[24] Obuchowski NA and Bullen JA (2018) Receiver operating characteristic (ROC) curves: review of methods with applications in diagnostic medicine. Physics in Medicine and Biology 63, 07TR01. 10.1088/1361-6560/aab4b1 29512515

[25] Mandrekar JN (2010) Receiver operating characteristic curve in diagnostic test assessment. Journal of Thoracic Oncology 5, 1315–1316. 20736804 10.1097/JTO.0b013e3181ec173d

